# Succinate Promotes Phagocytosis of Monocytes/Macrophages in Teleost Fish

**DOI:** 10.3389/fmolb.2021.644957

**Published:** 2021-04-15

**Authors:** Dai-xiao Yang, Hao Yang, Yun-chao Cao, Ming Jiang, Jun Zheng, Bo Peng

**Affiliations:** ^1^Center for Proteomics and Metabolomics, State Key Laboratory of Biocontrol, Guangdong Key Laboratory of Pharmaceutical Functional Genes, School of Life Sciences, Sun Yat-sen University, Guangzhou, China; ^2^Laboratory for Marine Biology and Biotechnology, Qingdao National Laboratory for Marine Science and Technology, Qingdao, China; ^3^Southern Marine Science and Engineering Guangdong Laboratory, Zhuhai, China; ^4^Faculty of Health Sciences, University of Macau, Macau, China

**Keywords:** monocytes/macrophages, metabolic regulation, innate immunity, aquatic pathogens, phagocytosis, succinate

## Abstract

Development of immunity-based strategy to manage bacterial infection is urgently needed in aquaculture due to the widespread of antibiotic-resistant bacteria. Phagocytosis serves as the first line defense in innate immunity that engulfs bacteria and restricts their proliferations and invasions. However, the mechanism underlying the regulation of phagocytosis is not fully elucidated and the way to boost phagocytosis is not yet explored. In this manuscript, we profiled the metabolomes of monocytes/macrophages isolated from Nile tilapia, prior and after phagocytosis on *Vibrio alginolyticus*. Monocytes/macrophages showed a metabolic shift following phagocytosis. Interestingly, succinate was accumulated after phagocytosis and was identified as a crucial biomarker to distinguish before and after phagocytosis. Exogenous succinate increased the phagocytotic rate of monocytes/macrophages in a dose-dependent manner. This effect was dependent on the TCA cycle as the inhibitor of malonate that targets succinate dehydrogenase abrogated the effect. Meanwhile, exogenous succinate regulated the expression of genes associated with innate immune and phagocytosis. In addition, succinate-potentiated phagocytosis was applicable to both gram-negative and -positive cells, including *V. alginolyticus, Edwardsiella tarda, Streptococcus agalactiae*, and *Streptococcus iniae*. Our study shed light on the understanding of how modulation on host’s metabolism regulates immune response, and this can be a potent therapeutic approach to control bacterial infections in aquaculture.

## Introduction

Bacterial infection accounts for huge economic loss in aquaculture (Meyer, 1991; Arkoosh et al., 1998; Pulkkinen et al., 2010). Antibiotics are routinely used to treat and prevent bacterial infections. However, the misuse of antibiotics selects or evolves the antibiotic-resistant bacteria. The treatment of infection by antibiotic-resistant bacteria requires higher dose or different types of antibiotics, which worsens the situation. More importantly, the misuse of antibiotics contaminates the environment and ultimately affects food quality and harms human health (Capita and Alonso-Calleja, 2013). In addition to chemical reagents, immunoprophylaxis constitutes another important measure to maintain sustainable aquaculture (Yang et al., 2013). Vaccine, for example, plays a critical role in preventing infectious diseases, including those caused by bacteria. They are highly specific and efficacious in prevention of disease. AQUAVAC^®^ Vibrio contains inactivated *Vibiro anguillarum* (biotype I and II) to grant protection of rainbow trout and European sea bass to vibriosis with strong immune response and broad-spectrum protection (Holten-Andersen et al., 2012). Various types of vaccines, such as subunit vaccine (Liu et al., 2020; Madusanka et al., 2020), DNA vaccine (Sun et al., 2010), and attenuated live vaccine (Buchanan et al., 2005; Locke et al., 2008; Coelingh et al., 2015), have been reported. However, most of the currently available vaccines in aquaculture only protect the hosts from limited strains of bacteria, whereas the complex ecosystem contains diverse types of pathogens that need to be dealt with. In addition, successful development of a vaccine is costly and time-consuming. Therefore, a novel strategy is urgently needed.

Boosting the innate immune is a promising approach. Being the same as in higher vertebrate, innate immunity is the most critical part in host defense system to pathogens in teleost (Riera Romo et al., 2016; Smith et al., 2019), featured by their acute and non-specific response. Hijacking the broad specificity of innate immune response may shed light on the development of novel strategies to manage bacterial infection (Kar and Joosten, 2020). Macrophage, one of the most important innate immune cells bridging innate immunity and adaptive immunity, plays a critical role in tight regulation of immune response through sequential secretion of immune mediators to control the immune response to an appropriate level (Rajaram et al., 2014; Oishi and Manabe, 2018). Upon bacterial infection, macrophage recognizes the pathogen-associated molecular patterns (PAMP) through surface receptors for phagocytosis that subsequently secretes a series of cytokines to promote inflammation, such as IL-1β, IL-6, IL-18, IFN-γ, TNF-α, and IL-8, which subsequently recruits other types of immune cells to eliminate infection at the site of infection (Garcia-Weber et al., 2018; Jung et al., 2020). Thus, modulating macrophage phagocytosis could be a way to enhance the clearance of bacterial infection, which is still lacking.

Metabolism has been considered to be the guiding force for immune response (Chen et al., 2017) and this idea was also proposed in aquatic animals. By profiling the metabolomes of the host at different biological conditions, one can harness the crucial biomarker and reprogram the metabolome to reinforce host’s ability to fight against infections and to eliminate antibiotic-resistant bacteria (Peng et al., 2015a). By comparing the metabolomes of dying fish and survival fish, we and other groups have shown that supplementation of zebrafish or tilapia with glucose, stearic acid, proline, serine, L-aspartic acid, or L-leucine could regulate the expression of immune-related genes and eventually increase fish’s survival upon the infection by *Vibrio alginolyticus*, *Streptococcus iniae, Streptococcus agalactiae*, or *Edwardsiella tarda* (Peng et al., 2015b; Du et al., 2017; Cheng et al., 2019). It was also revealed that tryptophan increased zebrafish survival to *V. alginolyticus* infection by downregulating the production of ROS to avoid excessive immune response (Gong Q. Y. et al., 2020). In addition, metabolism-based approach also found that maltose, palmitic acid, threonine, and taurine enhanced zebrafish survival to *Aeromonas sorbrial* or *E. tarda* infection at higher temperature (Jiang et al., 2019a). The supplementation of zebrafish with phenylalanine and maltose enhanced host’s capability to clear antibiotic-resistant bacteria that otherwise persist longer than antibiotic-sensitive bacteria in the host (Jiang et al., 2019b, 2020). Moreover, metabolite like glycine modulates *E*scherichia *coli* and *V. alginolyticus* metabolism to render them susceptible to serum in different species (Cheng et al., 2019). These studies suggest that metabolite-based regulation could be a potential strategy to combat bacterial infection through certain mechanism, for example, phagocytosis. With the aim of identifying metabolite to potentiate phagocytosis, we performed the functional metabolomics to compare the metabolome of fish-derived monocytes/macrophages before and following phagocytosis to *V. alginolyticus*. Succinate was identified as a crucial biomarker that was increased following phagocytosis. Exogenous administration of succinate enhanced phagocytosis through the citric acid cycle (TCA cycle) by increasing the expression of immune genes and phagocytic genes.

## Materials and Methods

### Fish Species and Bacterial Strains

Nile tilapia (*Oreochromis niloticus*) were obtained from Guangdong Tilapia Breeding Farm (Guangzhou, China) with equal number of male and female. The weight of each fish was around 500 ± 10 g. These animals were maintained in 25 L tanks under the following conditions as previously described (Du et al., 2017): water temperature at 28°C, pH value of 7.0–7.5, carbon dioxide <10 mg/L, oxygen at 6–7 mg/L, nitrogen content at 1–2 mg/L. Tilapia were acclimated for two weeks.

Bacterial strains used in this study include *V. alginolyticus* V12G01, *E. tarda* EIB202, *S. agalactiae*, and *S. iniae* (Zhao et al., 2015; Du et al., 2017; Peng et al., 2017; Yang J. et al., 2018). All of the strains except that *E. tarda*, a generous gift from Dr. Xiaohua Zhang of Ocean University of China, are preserved in our laboratory stocks. *V. alginolyticus* were grown in LB medium plus 3% sodium chloride; *E. tarda* were grown in TSB rich medium; *S. agalactiae* and *S. iniae* were grown in BHI medium. Bacteria were cultured at 37 and 30°C as indicated in the text for overnight with shaking. Overnight bacterial culture was reinoculated at a ratio of 1:100 until 1.0 at OD600, and bacteria were then washed three times with 1× PBS and finally resuspended in the 1× PBS.

### Isolation of Monocytes/Macrophages From Head Kidney

Isolation of macrophages from tilapia head kidney were performed as previously described (Mu et al., 2019). The head kidneys of tilapia were removed, grinded and the resultant homogenates were filtered through an 80 μm sterile steel mesh, followed by centrifugation and resuspension in L-15 medium supplemented with 10% fetal bovine serum (FBS) (Gibco, United States). Penicillin/streptomycin were added to a final concentration at 1% (Sigma, United States). Then, cells were layered on the top of the 54/31% Percoll (Sigma, United States), followed by centrifugation at 4°C. Cells at interface were carefully removed, and placed in a T25 flask, and incubated at 25°C for 24 h. The floating cells were removed, and the adherent cells were collected as the macrophages for the studies. Monocytes/macrophages cells were stained with Wright-Giemsa staining before the functional study as previously described (Mu et al., 2018; Zhang et al., 2018).

### Preparation of GC-MS Sample

Phagocytosis were performed as previously described (Chen et al., 2017). Here, we used *V. alginolyticus* for phagocytosis as this strain was avirulent to tilapia (data not shown) so that the metabolic shift by host-pathogen interaction can be excluded. Briefly, cells were cultured overnight in serum-free (0.5% FBS) medium and then incubated alone or together with *V. alginolyticus* cells at a multiplicity of infection of 100 in an incubator at 25°C for 1.5 h. Then, supernatants were carefully removed, and ice-chilled 1× PBS were added to stop the reaction. The cells were then quickly washed with 1× PBS by three times to remove any extracellular residual bacteria. After the last washing, ice-chilled methanol was immediately added to quench the bacterial metabolism, and total metabolites were extracted after adding 1 μg ribitol (Sigma, United States) as internal standard. The cells were lysed by ultrasonication, followed by centrifugation. Supernatants were collected and dried by vacuum.

### Gas Chromatography-Mass Spectrometry

Gas chromatography-mass spectrometry (GC-MS) sample were prepared as previously described (Jiang et al., 2020). Methoxyamine hydrochloride (80 μL of 20 mg/mL) (Sigma, United States) were added to the dried extracts, followed with 80 μL N-methyl-N-(trimethylsilyl) trifluoroacetamide (MSTFA, Sigma, United States). The mixture was then incubated at 37°C for 30 min for derivatization. The derivatized samples were centrifuged and supernatants were collected. 1 μL of the supernatant was transferred into a 30 m × 250 μm i.d. × 0.25 μm DBSMS column via Agilent autoinjector by splitless injection. The following parameters were set for GC-MS oven: the initial temperature: 85°C; hold: 5 min; heating rate: 15°C/min; the final temperature: 270°C and hold for 5 min. Helium is the carrier gas, whose flow rate is 1 mL/min. For mass spectrometry, electron impact ionization was set to 70 eV, and full scan mode was adopted which covers m/z 50–600.

### Data Processing

Date processing was conducted as previously described (Ma et al., 2015; Liu et al., 2019). Metabolites were identified in National Institute of Standards and Technology (NIST) (NIST MS search 2.0.) through the peaks of total ion chromatograms (TIC) in GC-MS. The metabolites were selected against the hits rate according to the ranking and matching factors (score: 999–600). The abundance of metabolite was determined by peaks area from TIC using XCalibur software (Thermo fisher, version 2.1). Silanization reagent from the data matrix were removed. Finally, the internal controls and total peak area were used to normalize the data matrix, which was set as external control. The ratio of the average intensity of ribitol to the individual intensity of ribitol was used as a factor for internal control-based normalization. In addition, the total peak area in the spectra were scaled to the average peak area of all sample spectra as the total area-based normalization. Data were normalized in the Microsoft Excel 2010.

### Multivariate Data Analysis of GC-MS Data

Metabolites with value of significant difference were identified through Manne–Whitney test (Wilcoxon rank sum test) and Kruskal–Wallis (KW) test in SPSS 13.0. Statistical analysis was performed by two-sided test, and is considered as statistical significance when *p* < 0.05. *Z-score* was adopted to analyze distribution of the experimental data to the mean, which was plotted based on the mean and standard deviation. Accordingly, monocytes/macrophages incubated with *V. alginolyticus* (Following-Phagocytosis, FP) was centered by monocytes/macrophages incubated with PBS (Before-Phagocytosis, BP) Orthogonal partial least squares discriminant analysis (OPLS-DA) was adopted to identify phagocytosis-associated patterns, and meanwhile that minimize the impact of differences among individual group (SIMCA 12.0 software) (Umetrics, Umeå, Sweden).

### Bioinformatics Analysis

The most relevant way is found with the online tool MetaboAnalyst 2.0. Overrepresentation is used to test whether the compounds participating in the pathway represent a larger number than random hits, and are performed with hypergeometric testing.

### Phagocytosis Assay

To assess the effects of succinate on phagocytosis, flow cytometry was adopted as described previously (Gong Q. et al., 2020). Briefly, monocytes/macrophages were seeded in a 6-well plate with 5 × 10^6^ cells at each well for overnight in serum-free medium. The cells were treated with ethyl succinate (0,5,10,20,40 mM) or diethyl malonate inhibitor (200 μM, Sigma, United States) in L-15 growth medium for 4 h. The untreated cells were served as a negative control. Then, FITC conjugated *V. alginolyticus* were added at an MOI at 100:1, briefly centrifuged, and incubated at 25°C for 1.5 h (Du et al., 2017). The plates were washed with ice-chilled 1× PBS to stop reaction. Phagocytosis was analyzed in a flow cytometer. Three replicates were performed for each treatment, and 10,000 cells were gated for each sample. The percentage of monocytes/macrophages that phagocytosed at least one type of bacteria in the total monocyte/macrophage population is taken as the phagocytosis rate. Phagocytic index is defined as the average number of bacteria in phagocytic cells which was quantified in flow cytometer by the mean channel of fluorescence (Juan et al., 2018). In order to test the universality of the phagocytosis effect of succinate on monocytes/macrophages, gram-negative or gram-positive bacteria including *E. tarda*, *S. agalactiae*, and *S. iniae* were selected for phagocytosis *in vitro*. The changes of phagocytosis were detected by flow cytometry.

### Enzymatic Activity Assay

Enzymatic activities were quantified as previously described (Liu et al., 2019). Briefly, monocytes/macrophages were treated with 20 mM ethyl succinate or left untreated. Four biological replicates were included in each group. Protein concentration were measured with bicinchoninic acid assay (Beyotime Biotechnology). The activity of pyruvate dehydrogenase (PDH) and α-ketoglutarate dehydrogenase (αKGDH) were measured in the following reaction that includes 0.15 mM MTT, 0.5 mM PMS, 0.2 mM TPP, 2.5 mM MgCl2, 50 mM potassium phosphate buffer, 2 mM pyruvate or 5 mM α-ketoglutarate potassium salt at pH 7.0. The activity of succinate dehydrogenase (SDH) and malate dehydrogenase (MDH) was measured in following reaction that include 0.15 mM MTT, 1 mM PMS, 50 mM potassium phosphate buffer, 20 mM succinate or 50 mM malate at pH 7.0. 200 μg of total protein were added to the corresponding system to a final volume of 200 μL in a 96-well plate. The plate was incubated at 37°C for 20 min for KGDH and MDH, or 8 min for SDH and PDH. MTT, PMS, TPP, pyruvate, succinate, malate, and α-ketoglutaric acid potassium salt were purchased from Sangon Biotech.

### Quantitative Real-Time PCR

Quantitative real-time PCR (qRT-PCR) was performed as previously described (Tang et al., 2007; Wang Y. et al., 2019). The total RNA of monocytes/macrophages was extracted in Trizol (Invitrogen, United States), and quantified by fluorescence intensity detection. Before reverse transcription, gDNA was eliminated by gDNA eraser. qRT-PCR was conducted with PrimeScript^TM^ RT reagent Kit (AG, China) with 1 μg of total RNA. cDNAs were synthesized with reverse transcription kit, and used as the template for qRT-PCR. The cDNA was then used for qPCR and the reaction was performed in 384-well plates that includes 5 μL 2 × SYBR Green *Pro Taq* HS Premix, 1.3 μL H_2_O, 3.3 μL cDNA, and 0.2 μL of each primer (10 μM). qRT-PCR was run on CFX384 Touch (Bio-Rad, United States) as following: 95°C for 30 s, 40 cycles for 10 s at 95°C, extended at 56°C for 30 s. Fluorescence measurement was taken at 72°C at each cycle. The melting curve is obtained when the cycle at 95°C that has a calefactive velocity at 5°C/s. Use 2^−ΔΔct^ method to normalize the expression of samples by β*-actin*, *gapdh*, and *ef1*α (Livak and Schmittgen, 2001). All primers are listed in Supplementary Table 1.

## Results

### Metabolomic Profiling of Monocytes/Macrophages Before and Following Phagocytosis

**FIGURE 1:**
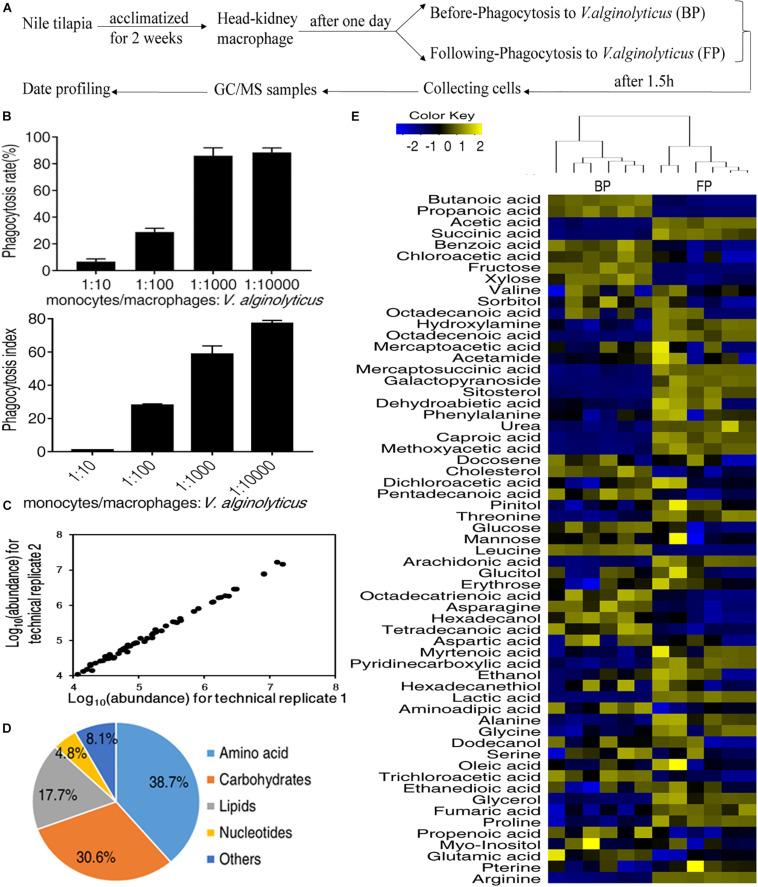
Metabolic profiles of monocytes/macrophages before and following phagocytosis. **(A)** Schematic representation of experiment workflow; **(B)** The phagocytosis rate and phagocytic index of monocytes/macrophages. **(C)** Reliability of metabolomic profiling for the replicates. Pearson correlation coefficient was shown. **(D)** Functional categories of the metabolites. **(E)** Heat map of all metabolites (row). Yellow indicates the increase in the abundance of metabolites and blue indicates decrease.

To investigate the role of metabolic pathways that control fish macrophage phagocytosis, we isolated the primary monocytes/macrophages from head kidneys of Nile tilapia, *O. niloticus*, to perform a systematic metabolomic analysis. The process was outlined in Figure 1A. The morphology and purity of isolated macrophage was also checked by Wright-Giemsa staining (Supplementary Figure 1). Macrophages showed a phagocytosis rate of 28.99 ± 2.72%, and the phagocytic index was 27.90 ± 0.96 to FITC-labeled *V. alginolyticus* at a MOI of 1:100 as quantified by flow cytometry (Figure 1B). Therefore, macrophages incubated with either *V. alginolyticus* (Following-Phagocytosis, FP) or 1× PBS (Before Phagocytosis, BP) were subjected to GC-MS analysis. There were three biological replicates and two technical replicates for each treatment. The reliability of GC-MS was evaluated by the correlation coefficient of two technical replicates (Figure 1C).

A total of 62 metabolites were identified for each treatment. According to the functional categorization, 38.7% of the metabolites were amino acids, 30.6% of the metabolites were carbohydrates, 17.7% of the metabolites were lipids, 8.1% of the metabolites were nucleotides, and 4.8% of the metabolites were others (Figure 1D). The relative abundance of the identified metabolites was shown in Figure 1E. The heatmap showed that the replicates of BP and FP were clustered together, indicating that BP and FP have distinct metabolism (Figure 1E).

### Differential Metabolomic Profiling of Monocytes/Macrophages Following Phagocytosis

**FIGURE 2:**
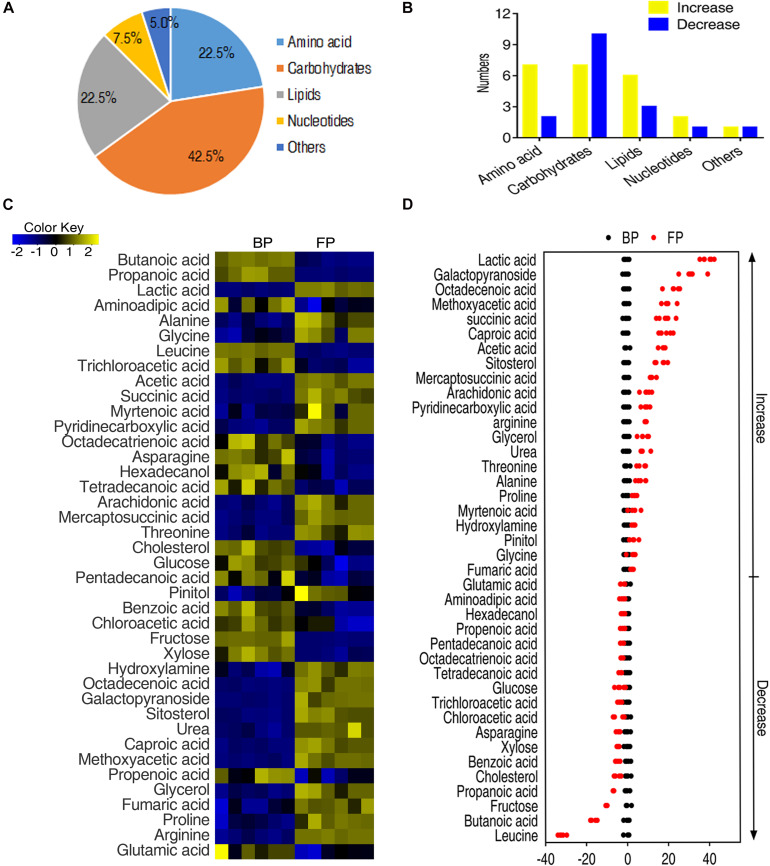
Metabolic profiling of monocytes/macrophages of *O. niloticus* before and after phagocytosis. **(A)** Functional categories of the differential metabolites. **(B)** Histogram of altered metabolite of each functional category after phagocytosis. **(C)** Changes of differential metabolites in tilapia head kidney monocytes/macrophages following phagocytosis of bacteria by Wilcoxon, *p* < 0.01). **(D)** Z-score plots of differential metabolites of tilapias head kidney monocytes/macrophages following phagocytosis of bacteria.

To identify the metabolites that were contributing to distinguish BP and FP, the abundance of metabolites between the two groups was tested for statistical differences using two-sided Wilcoxon rank sum test and permutation test. Among the identified 62 metabolites, the abundance of 40 metabolites was significantly altered. The differential metabolites fell to the functional categories of carbohydrates, amino acids, lipid nucleotides, and others (Figures 2A,B). The change in the abundance of metabolites were analyzed by unsupervised hierarchical clustering (Figure 2C). Moreover, the changes in the abundance of metabolites in the two groups can be represented by the *Z-score* (Figure 2D), where 22 metabolites, such as lactic acid, glucopyranoside, octadecenoic acid, were elevated and 18 metabolites, such as leucine, butanoic acid and fructose, were decreased in FP. The *Z-score* of the FP group ranges from −32.09 to 39.76 for a comparative study (Figure 2D). These data suggest that the monocytes/macrophages form certain metabolic pattern after phagocytosis.

### Pathway Analysis of Metabolites With Differential Abundance

**FIGURE 3:**
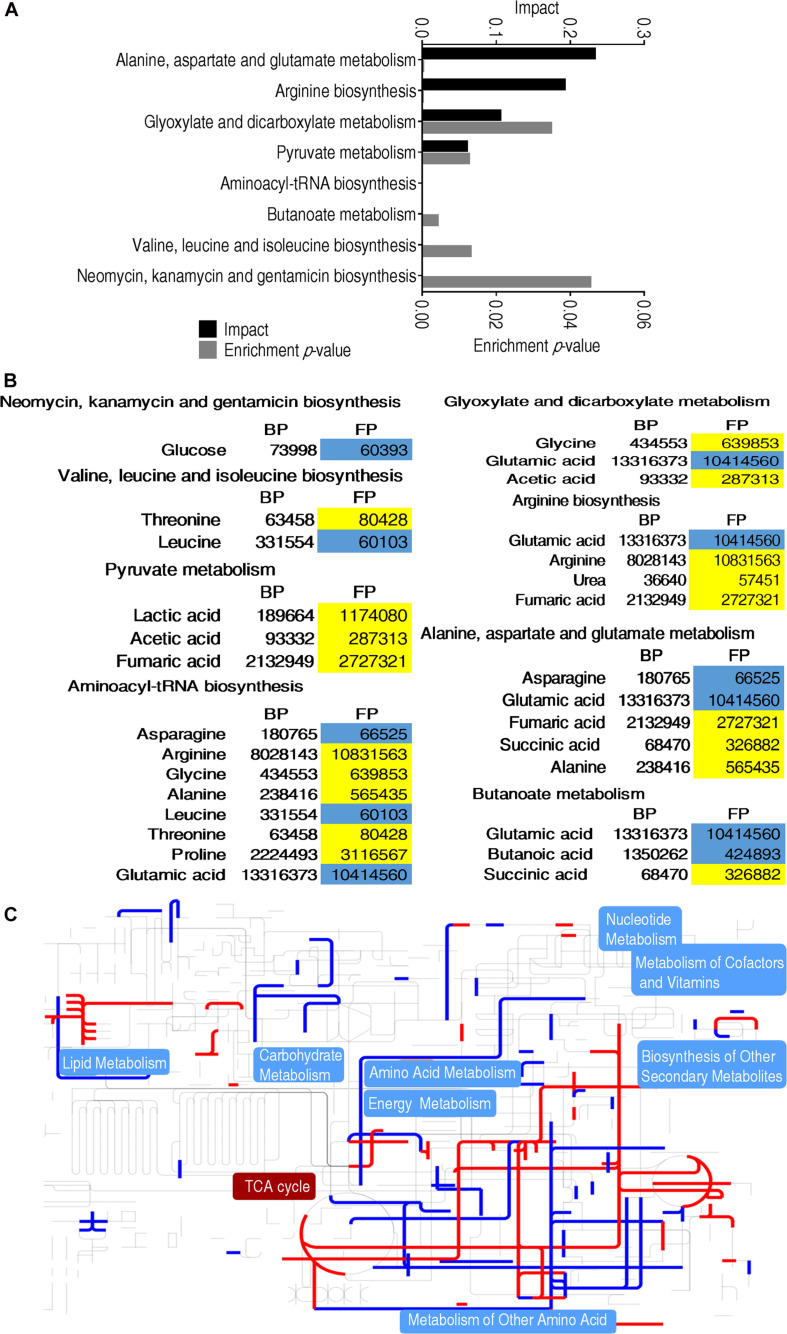
Metabolomic alterations at different groups. **(A)** Pathway enrichment analysis of differential metabolites that were used for visualization. **(B)** Heat map of differential metabolites. Yellow indicates an increase in metabolites and blue indicates a decrease, which were represented by the average and standard deviation of the metabolite relative abundance. **(C)** iPath analysis of the differential metabolites.

Metabolites of differential abundance between BP and FP were further analyzed by pathway enrichment, and finally a total of eight metabolic pathways were enriched. The first top four pathways were aminoacyl-tRNA biosynthesis, alanine, aspartate and glutamate metabolism, arginine biosynthesis, pyruvate metabolism, respectively (Figure 3A). These four pathways were strongly associated with amino acid metabolism and carbohydrates metabolism. On the other hand, all metabolites detected were elevated only in pyruvate metabolism. Most of the metabolites of the pathways were upregulated in FP group, indicating that monocytes/macrophages enhance amino acid metabolism and carbohydrates metabolism for phagocytosis (Figure 3B). Furthermore, iPath was adopted to generate a global view of the differential metabolites, which showed the upregulated central carbon metabolism, especially pyruvate metabolism and the TCA cycle (pyruvate metabolism provides source for the TCA cycle) (Figures 3B,C), consistent with the increased succinate and fumarate of the TCA cycle (Figure 3C).

### Multivariate Data Analysis

**FIGURE 4:**
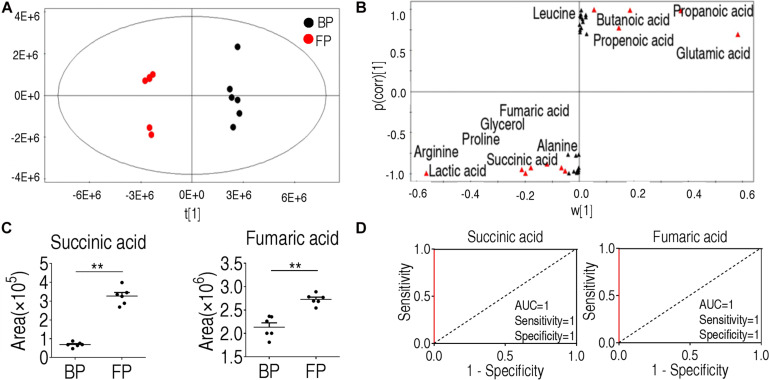
Identification of crucial biomarkers before and after phagocytosis. **(A)** OPLS-DA analysis of metabolic data before and following phagocytosis. Each point represents a technical replicate of the sample. **(B)** S-plot. p [1] and correlation p(corr) [1] distinguish monocytes/macrophages before and after phagocytosis. **(C)** Succinate and fumarate were the crucial biomarkers for phagocytosis. **(D)** ROC curve of biomarkers for phagocytosis. *p < 0.05; **p < 0.01.

Orthogonal Partial Least Squares Discriminant Analysis was then applied to identify the metabolites that are critical for phagocytosis. The component (*t*[1]) clearly separated the two groups of BP and FP (Figure 4A). The absolute values of covariance p and correlation p (corr) were set to be greater than or equal to 0.05 and 0.5, respectively (Figure 4B). As shown in Figure 4C, each red triangle represented an individual crucial biomarker to distinguish the BP and FP groups. The twelve biomarkers included alanine, succinate, lactic acid, leucine, butanoic acid, fumarate, glycerol, proline, propanoic acid, propenoic acid, glutamic acid, and arginine (Figure 4C and Supplementary Figure 2A). To convert the crucial biomarkers into a predicting model of monocytes/macrophages phagocytosis, ROC curve was adopted and shown in Figure 4D and Supplementary Figure 2B. Succinate, fumarate, propanoic acid, arginine, glycerol, glutamic acid, lactic acid showed the most significant value that was greater than 0.9 for the AUC value, implying that these metabolites form a metabolic signature of phagocytosis. Out of the twelve metabolites, succinate and fumarate, the key intermediates of the TCA cycle and pyruvate metabolism, respectively, were the most crucial biomarkers in differentiating the FP and BP groups.

### Exogenous Succinate Upregulates Phagocytosis in Monocytes/Macrophages

**FIGURE 5:**
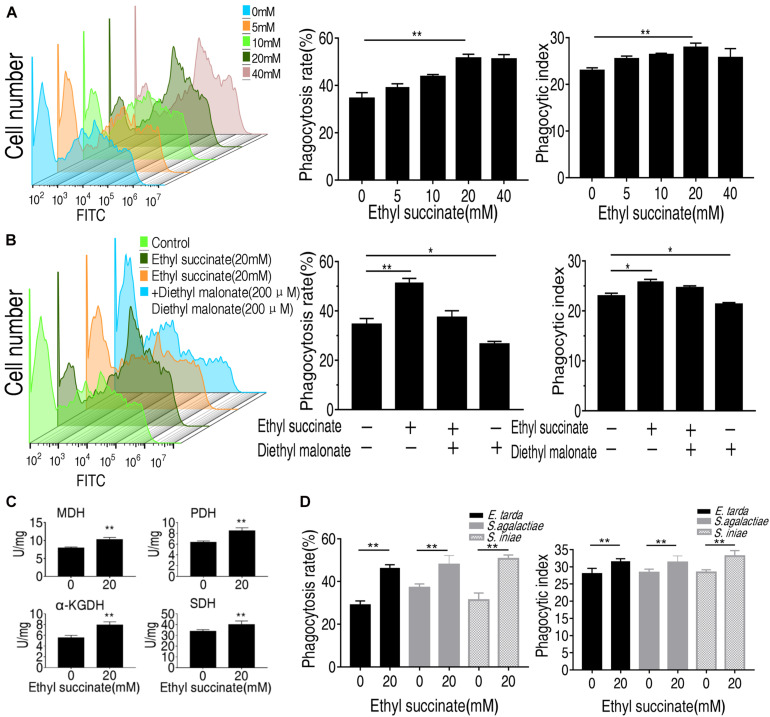
Succinate promotes the phagocytosis of *O. niloticus* monocytes/macrophages. **(A)** the monocytes/macrophages were incubated with or without ethyl succinate (20 mM) for 4 h. **(B)** the monocytes/macrophages were incubated with or without succinate or diethyl malonate treatment for 4 h. **(C)** the monocytes/macrophages activity of PDH, MDH, KGDH, and SDH. **(D)** Phagocytosis rate and phagocytic index of FITC-conjugated gram-negative or gram-positive bacteria by succinate-pretreated monocytes/macrophages at indicated concentration. Error bars were represented as means ± SEM from three biological replicates. Statistical significance was obtained when *p < 0.05 or **p < 0.01.

Succinate was used for subsequent analysis since its abundance was increased at least two-folds following phagocytosis. It belongs to the TCA cycle. Another crucial TCA metabolite, fumarate, has less significant changes in abundance than succinate (Figure 4C), and thus was not subject to further investigation. Exogenous succinate increased phagocytosis and phagocytic index in a dose-dependent manner (Figure 5A). To investigate whether succinate promotes phagocytosis through the TCA cycle, malonate was used to inhibit the SDH and both the normal phagocytosis and succinate-enabled phagocytosis decrease accordingly (Figure 5B). Similarly, the phagocytic index was also decreased after SDH inhibition (Figure 5B). Of note, inhibition of SDH alone also decreased phagocytosis and phagocytic index, implying succinate catabolism is critical for phagocytosis (Figure 5B). The data were consistent with the observation that the activity of α-KGDH, PDH, SDH, and MDH were increased after being treated with exogenous succinate (Figure 5C). In addition, the role of succinate in promoting monocytes/macrophages phagocytosis and phagocytic index were also shown in other pathogenic bacteria including *E. tarda*, *S. agalactiae*, and *S. iniae* (Figure 5D). These results suggest that succinate enhances monocytes/macrophages of phagocytosis via TCA cycle.

### Succinate Enhances Immune Response in Monocytes/Macrophages

**FIGURE 6:**
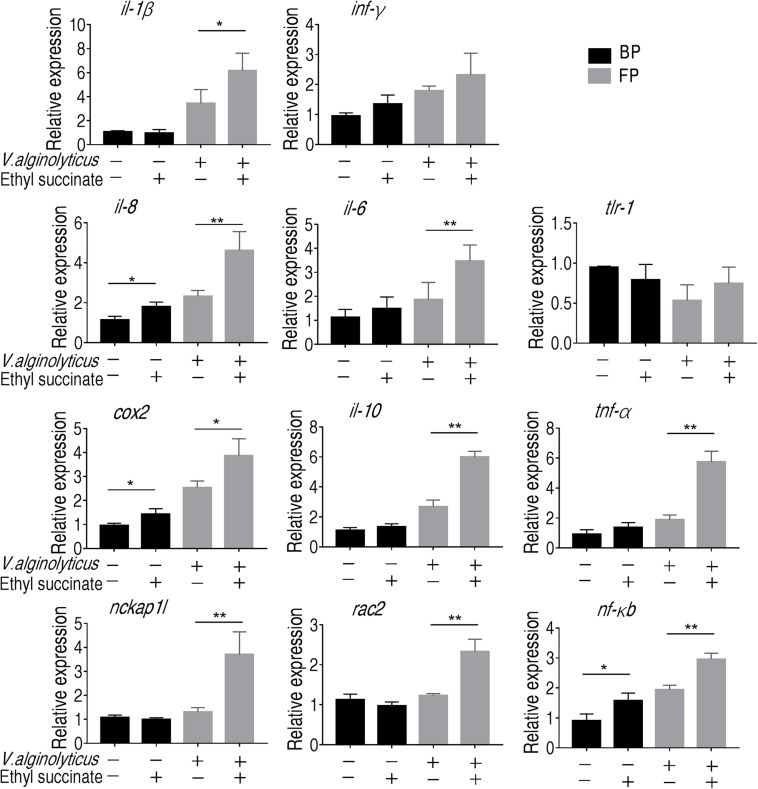
Gene expression of monocytes/macrophages. qRT-PCR of the immune genes and phagocytic genes before and after phagocytosis. Error bars were represented as means ± SEM from three biological replicates. Statistical significance was obtained when *p < 0.05 or **p < 0.01.

Macrophage eliminates and destroys invaders by phagocytizing the invaders and secreting cytokines that recruit other immune cells to the site of infection. To further demonstrate that succinate plays a role in the promotion to monocytes/macrophages and explore a possible reason, we measured the transcriptional levels of the immune-related genes (*tnf-*α, *il-8*, *il-1b*, *il-6*, *ifn-*γ, *cox-2*, *tlr-1*, *nf-*κ*b*, and *il-10*) and two phagocytic genes (*rac2* and *nckap1l*). Rac family small GTPase 2, encoded by *rac2* gene, are critical for phagocytosis and cell motility in macrophage (Tell et al., 2012; Collins et al., 2015; Haney et al., 2018). Nck-associated protein 1-like, encoded by *nckap1l*, is highly conserved from invertebrates to mammals (Baumgartner et al., 1995). The deficiency in *nckap1l* caused anomalies in lymphocyte development, phagocytosis and neutrophil migration in zebrafish and mouse (Tell et al., 2012; Haney et al., 2018; Castro et al., 2020). However, the expression level for all the genes except for *inf-*γ and *tlr-1* was boosted for at least two-folds in the presence of succinate following phagocytosis (Figure 6). These results indicate that succinate plays a crucial role in promoting phagocytosis and subsequent immune response.

## Discussion

Elimination of bacterial infection is critical for the sustainable development in aquaculture. Misuse of antibiotics in aquaculture raised concerns about the selection or generation of drug-resistant bacteria. In addition, the residual antibiotics in aquatic animal present potential risk for food safety. Thus, policies to restrict the use of antibiotics have been by governments throughout the world (Ajibola et al., 2018). Vaccines and immunomodulators are of great interest in modulating host’s immunity for combating bacterial infection (Thakur et al., 2019). The shortage of commercially available vaccines for aquaculture challenges this strategy. Under such circumstance, it is essential to develop a metabolic strategy to boost the immune system that can be widely adopted in different aquatic animals.

In this study, we present a metabolic approach to enhance the phagocytosis of the monocytes/macrophages, which is largely unexplored in aquaculture. Conditions being applied to isolation of monocytes/macrophages varied among different fish species, e.g., by Ficoll (1.077 g/ml) in ayu, *Plecoglossus altivelis* (Zhang et al., 2018) and by 34/51% Percoll in large yellow croaker (Li et al., 2019). We took a previously established protocol (Mu et al., 2019) and obtained good yields of healthy monocytes/macrophages (Supplementary Figure 1). But it is worthy to try other conditions that might give higher yields. Followed with functional metabolomics, we uncovered that monocytes/macrophages adjusts their metabolisms following phagocytosis. The adjusted amino acid metabolism and central carbon metabolism form the characteristic feature of monocytes/macrophages following phagocytosis. L-proline, L-arginine and L-alanine, for example, were increased following phagocytosis. L-arginine, for example, has been well documented on its role in immune response in higher vertebrate. L-arginine can be *de novo* synthesized from L-proline (Jenkinson et al., 1996), and L-arginine can be catabolized by NOS, arginase (ARG), and L-arginine decarboxylase. ARG and NOS are important mediators of immune response by promoting the activation of monocytes/macrophages and the secretion of cytokines (Martinez et al., 2009; Grohmann and Bronte, 2010). The role of L-alanine on immunity was less explored, especially in aquatic animal. However, extracellular L-alanine was important for T-cell activation, and the deprivation of alanine at early activation leads to functional impairment of the T-cell (Ron-Harel et al., 2019). Therefore, whether these three metabolites also participate in phagocytosis requires further investigation.

Through the integrative analysis of the metabolite of differential abundance, we identified the TCA cycle being the central at phagocytosis. Disruption of the TCA cycle was shown to be a metabolic signature of monocytes/macrophages activation and immune response (Stunault et al., 2018), which lies at the hub of adjusting metabolic pathways. We have previously observed that TCA cycle were required to combat against *V. alginolyticus* infection but not to clear *E. tarda* infection (Zeng et al., 2017; Yang M. J. et al., 2018). This differential requirement of the TCA cycle to eliminate bacterial infection implied that distinct metabolisms are required for different pathogens.

Although the TCA cycle has long be thought as the powerhouse that provides energy for immune cell activation, it was only recently recognized that the intermediates of the TCA cycle were players in regulating immune response (Viola et al., 2019). Here, we found that succinate was critical for the controlling of phagocytosis. Exogenous methyl-succinate could enhance phagocytosis, pro-inflammatory cytokine production and expression of phagocytic genes. This result is consistent with previous finding that succinate regulates the stability of hypoxia-induced factor-1α (HIF-1α) through inhibition of prolyl hydroxylases (PHD), leading to increased production of IL-1β (Tannahill et al., 2013). However, whether succinate links phagocytosis via HIF-1α requires further investigation. Fumarate is another identified intermediate metabolite in this study. The role of fumarate was less explored in immune response. However, it is shown that exogenous dimethyl fumarate is potent antioxidant and immunomodulators of immune response to alleviate excessive immune response (Kornberg et al., 2018). How it promotes phagocytosis is unknown.

To further explore the role of succinate on immune gene expression, we profiled the expression of genes that are commonly involved in inflammatory response. We have examined nine genes, namely *il-1b*, *inf-*γ, *cox-2*, *tlr-1*, *nf-*κ*b*, *il-8*, *tnf-*α, *il-6*, and *il-10* (Cheng et al., 2018). The pro-inflammatory cytokine IL-1β is produced by a variety of cells including immune cells (such as monocytes and macrophages), non-immune cells (such as keratinocytes), and some epithelial cells. IL-1β can promote inflammation through increasing the production of IL-2 and nitrogen oxide, and recruiting neutrophiles (Tsai and Tsai, 2017). IL-8 is both a pro-inflammatory and growth factor that acts as chemokine to recruit neutrophile and promote angiogenesis. IL-8 has been found to be associated with inflammatory diseases (Wang X. et al., 2019). The pro-inflammatory cytokine TNF-α exerts pleiotropic effects on a variety of cell types (Szondy and Pallai, 2017). IL-6 plays a role in inflammation and B cell maturation by activating Janus kinases (JAK) and signal transducers and activators of transcription (STAT). COX-2 is highly expressed by activated macrophages, and also in other inflammatory cells. It is an inducible cyclooxygenase and is known as an inflammatory molecule in fish (Moore et al., 1998). It’s not astonishing to find that the expression of these genes was upregulated. Interestingly, succinate also increases the expression of *il-10*. Although *il-10* is known to negatively regulate inflammation, it also exerts role in promoting phagocytosis that had been demonstrated both in human and fish (Capsoni et al., 1995; Yang et al., 2019). It is possible that succinate may induce an IL-10-dependent pathway for promoting phagocytosis in monocytes/macrophages in Nile tilapia. The expression of two cytokines, *inf-*γ and *tlr-1*, were not changed. A previous study showed that the leukocytes isolated from rainbow trout head kidney failed to upregulate *inf-*γ in response to the stimulus of LPS, flagellin, zymosan and β-glucan (Chettri et al., 2011). Thus, *V. alginolytius* might be a weak elicitor of *inf-*γ in tilapia as well. Toll-like receptor 1 (TLR-1) of *O. niloticus* is ortholog to that of other species including zebrafish, *Danio rerio* (Abouelmaatti et al., 2020). In *D. rerio*, the ligand for TLR1 is the TLR1-2 heterodimer, whereas TLR2 is known to recognize the dipeptides and Pam3CSk4 that is associated with Gram-positive bacteria (Pietretti and Wiegertjes, 2014; Li et al., 2017). As Gram-negative bacteria, *V. alginilyticus* failed to induce the activation of *tlr-1*. Interestingly, succinate alone had minor effects on the gene expression in the absence of bacteria except that the transcriptions of *il-8*, *cox-2*, and *nf-*κ*b* were increased 1.50–1.74-folds. But their expression was increased for more than two-folds once the bacteria were present. Thus, we postulate that succinate may have multiple targets other than HIF-1α, which are induced by the exogenous flux of succinate (Tannahill et al., 2013). It would be interesting to investigate whether such increase have functional consequence, e.g., increased cytokine production, should also be investigated in the future.

In conclusion, our data establish a novel approach to examine the metabolic pathways and identify metabolites that regulate the phagocytosis. Crucial biomarkers identified, such as succinate, could potentially promote the phagocytosis of monocytes/macrophages. This capability was applicable to a variety of pathogens, indicating a promising approach of using metabolite in aquaculture for combating bacterial infection. Thus, we present a metabolic-based screening of potential metabolites in promoting immune response to bacterial infection.

## Data Availability Statement

The datasets presented in this study can be found in online repositories. The names of the repository/repositories and accession number(s) can be found in the article/ Supplementary Material.

## Ethics Statement

All of the animal experiments performed in this study were reviewed and approved by the Institutional Animal Care and Use Committee of Sun Yat-sen University (Approval No. SYSU-IACUC-2020-B1267).

## Author Contributions

DY and HY conducted the experiments. DY, HY, and YC performed the data analysis. DY, HY, YC, JZ, and BP interpreted the data. BP wrote the manuscript and conceptualized and designed the project. All the authors reviewed the manuscript and acknowledged the contributions.

## Conflict of Interest

The authors declare that the research was conducted in the absence of any commercial or financial relationships that could be construed as a potential conflict of interest.
