# Supplementary figures and images for: Succinate Promotes Phagocytosis of Monocytes/Macrophages in Teleost Fish
Source: Front Mol Biosci. 2021 Apr 15;8:644957. doi: 10.3389/fmolb.2021.644957 (PMC8082191; doi:10.3389/fmolb.2021.644957)

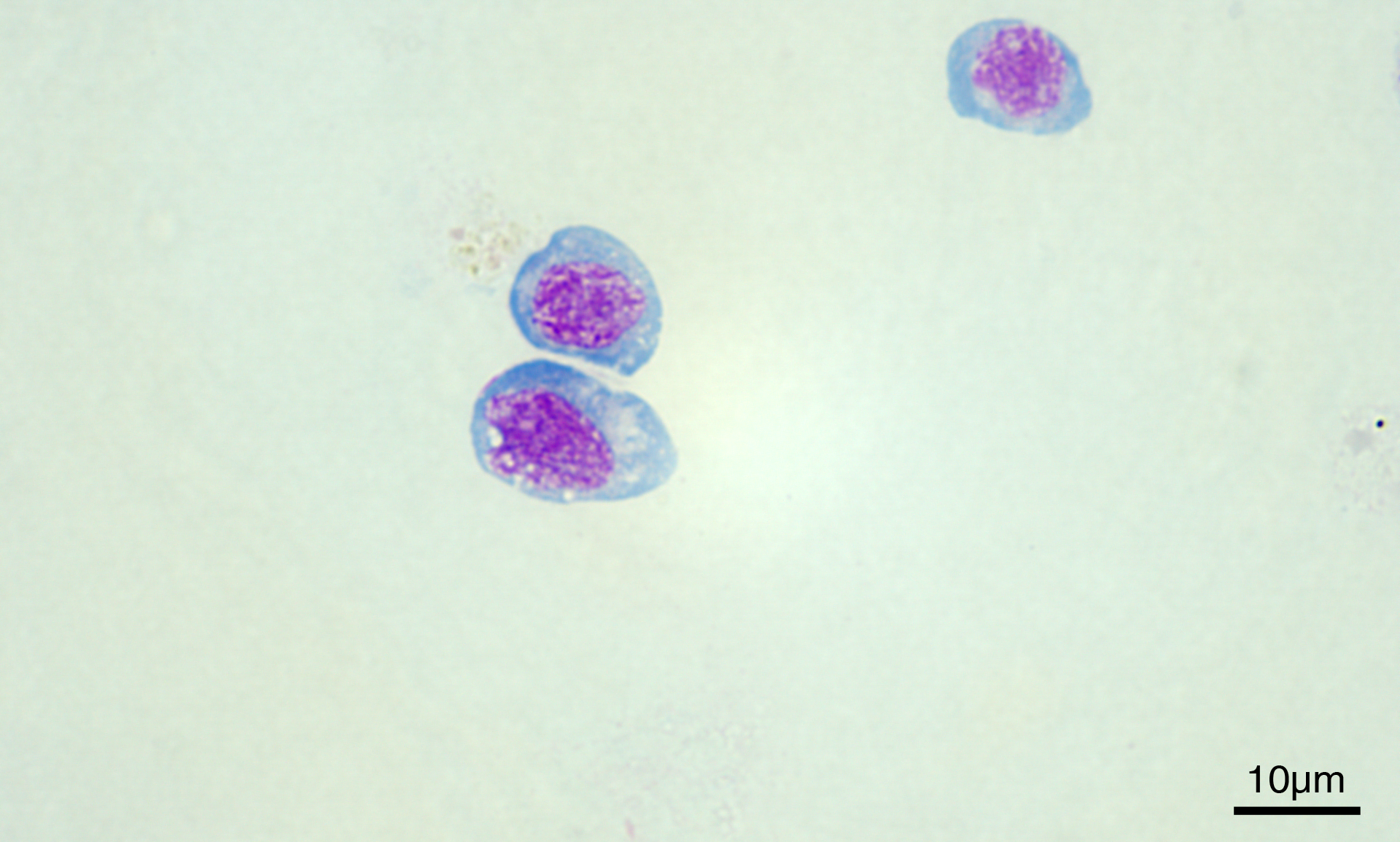

Supplement: Supplementary file 1 [file Image_1.TIF]

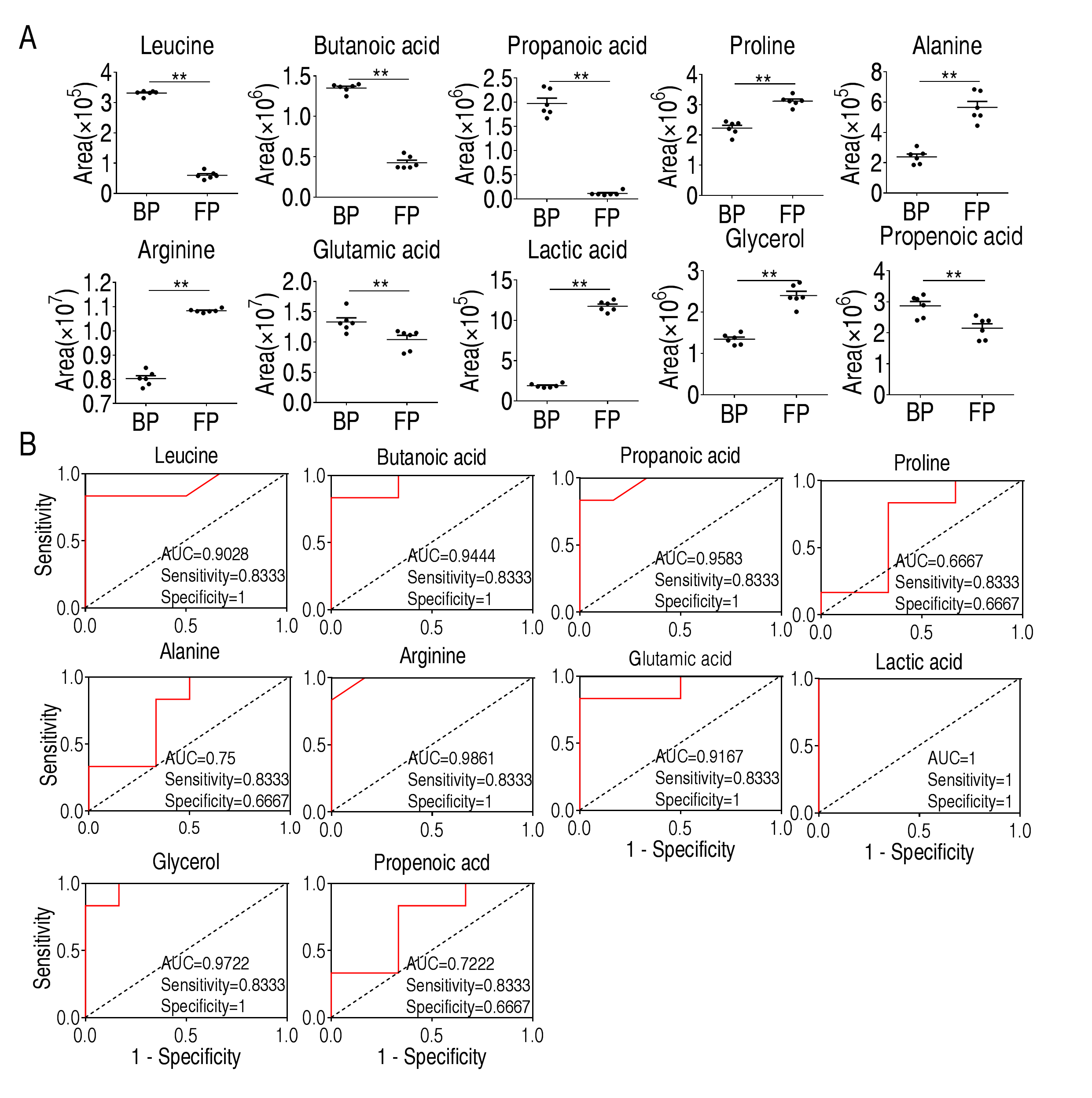

Supplement: Supplementary file 2 [file Image_2.TIF]
