# Supplementary material for: Succinate Promotes Phagocytosis of Monocytes/Macrophages in Teleost Fish
Source: Front Mol Biosci. 2021 Apr 15;8:644957. doi: 10.3389/fmolb.2021.644957 (PMC8082191; doi:10.3389/fmolb.2021.644957)
Supplement: Supplementary file 3 [file Table_1.docx]

**Supplementary Table**

Table 1 Primers used in this study.

| **Gene** | **Accession number** | **Primer** | **Nucleotide Sequence (5′-3′)** | |  |
| --- | --- | --- | --- | --- | --- |
| *β-**actin* | 100534414 | Forward | AATCGTGCGTGACATCAAAG |  |  |
|  |  | Reverse | GACGTCGCACTTCATGATG |  |  |
| *gapdh* | 100704894 | Forward | TTA AGG AAG CCG TCA AGA AG | | |
|  |  | Reverse | CAG CAC CAG CAT CAA AGA | | |
| *ef1α* | 100534431 | Forward | CTACGTGACCATCATTGATGCC | | |
|  |  | Reverse | AACACCAGCAGCAACGATCA | | |
| *il-1b* | 100693282 | Forward | GCGTGCCAACAGTGAGAA | | |
|  |  | Reverse | CAGGAGGGACGGAAGGGAT | | |
| *il-8* | 106098416 | Forward | GTATGCCGCCAATCAGCC | | |
|  |  | Reverse | GCTCCGTTTGCCAGTCCAG | | |
| *tnf-α* | 100534578 | Forward | GTCGTCGTGGCTCTTTGTTTAG | | |
|  |  | Reverse | GCCTTGGCTTTGCTGCTGAT | | |
| *il-6* | 100702023 | Forward | ATGCCTGGCGTTGAGTACCT | | |
|  |  | Reverse | CAAAATCGCTGACGTGATTGA | | |
| *tlr-1* | 102076024 | Forward | GAGACCGGACTGCACGGCTAT | | |
|  |  | Reverse | ACTCAGTTCCTTCCAGCGTTT | | |
| *inf-γ* | 100703830 | Forward | GCATCTGCCAATGTCTTCACAC | | |
|  |  | Reverse | GCTGCTGTTCTTGCCTTTACTG | | |
| *il-10* | 100694754 | Forward | CCAATCAGCCGTGACTACAACA | | |
|  |  | Reverse | GTGGAATGAGGGTTCAGACAAA | | |
| *nf-κb* | 100702187 | Forward | ATCAGAAATGCTGCCGCTATG | | |
|  |  | Reverse | CGGAGCCTTGCTGTCGTAGAT | | |
| *cox2* | 8677314 | Forward | GGGGATTCAACTGGAACTACTATTC | | |
|  |  | Reverse | AAGATCTCGGCTTCGATTCTTAT | | |
| *rac2* | 100695482 | Forward | CCATCATCCTGGTTGGCACTA | | |
|  |  | Reverse | GGGTCAAGGCTGAGCACTCC | | |
| *nckap1l* | 100692363 | Forward | GCCAAACTCCTCCGTGAGCT | | |
|  |  | Reverse | GACGAATGGAAGCAGTGACCC | | |
